# Hereditary gastrointestinal polyposis syndromes Rare Disease Collaborative Network consensus statement agreed at the RDCN meeting Birmingham 17th February 2022

**DOI:** 10.1038/s44276-023-00011-z

**Published:** 2023-08-04

**Authors:** Susan Clark, Vicky Cuthill, Jackie Hawkins, Warren Hyer, Andy Latchford, Ashish Sinha, Farhat Din, Andrew Beggs, Anant Desai, Dion Morton, Debbie Hitchen, James Hill, Fiona Lalloo, Katy Newton, Sarah Pugh, Sunil Dolwani, Rachel Hargest, James Horwood, Raji Ramaraj, Mark Rogers, Paul Collins, Frances McNichol, Nicholas Beck, Lucy Side, Frank McDermott

**Affiliations:** 1 The St Mark’s Centre for Familial Intestinal Cancer, St Mark’s Hospital, London North West University Healthcare NHS Trust, London, UK; 2 Department of Surgery and Cancer, Imperial College, London, UK; 3 Institute of Genetics and Cancer, The University of Edinburgh, Western General Hospital Campus, Edinburgh, UK; 4 Institute of Cancer and Genomic Sciences, College of Medical and Dental Science, University of Birmingham, Birmingham, UK; 5 Department of Surgery, Queen Elizabeth Hospital, Birmingham, UK; 6 Department of Colorectal Surgery, Central Manchester University Hospitals NHS Foundation Trust, Manchester, UK; 7 Clinical Genetics Service, Manchester Centre for Genomic Medicine, Central Manchester University Hospitals NHS Foundation Trust, Manchester, UK; 8 Division of Population Medicine, School of Medicine, Cardiff University, Cardiff, UK; 9 Department of Colorectal Surgery, University Hospital of Wales, Cardiff, UK; 10 Department of Gastroenterology, University Hospital Llandough, Cardiff, UK; 11 Department of Medical Genetics, Cardiff University, Cardiff, UK; 12 Department of Gastroenterology and Hepatology, Royal Liverpool University Hospital, Liverpool, UK; 13 Department of Colorectal Surgery, Royal Liverpool University Hospital, Liverpool, UK; 14 Colorectal Unit, University Hospitals Southampton NHS Foundation Trust, Southampton, UK; 15 Wessex Clinical Genetics Service, University Hospital Southampton NHS Foundation Trust, Southampton, UK; 16 Royal Devon University HealthCare NHS Foundation Trust, Exeter, UK

## Introduction

NHS England set out in the *Implementation Plan for the UK Strategy for Rare Diseases* (https://www.england.nhs.uk/wp-content/uploads/2018/01/implementation-plan-uk-strategy-for-rare-diseases.pdf) that it would develop and implement Rare Disease Collaborative Networks (RDCNs), with the definition of a RDCN being a ‘recognised network of member providers, each of which has demonstrable research-active interest in a rare/very rare disease, the aim of the network being to improve patient outcomes’. The network is composed of Rare Disease Collaborative Centres. A Rare Disease Collaborative Centre (RDCC) is a ‘provider that has been recognised as having a demonstrable research-active interest in a rare/very rare disease and who works with other recognised providers in a network to improve patient outcomes’.

This initiative is also in line with the NHS England Board paper ‘12 Actions to Support and Apply Research in the NHS’ discussed on 30 November 2017 (https://www.england.nhs.uk/wp-content/uploads/2018/05/12-actions-to-support-and-apply-research-in-the-nhs.pdf).

## Conditions covered

- Familial adenomatous polyposis.
- *MutYH*-associated polyposis.
- Polymerase proofreading associated polyposis.
- Peutz-Jeghers syndrome.
- Juvenile polyposis syndrome.
- Other ultra-rare Mendelian polyposis syndromes.

## Rare Disease Collaborative Centres and Network

The following centres are now recognised by NHSE:

- Birmingham.
- Cardiff.
- Edinburgh.
- Manchester (with Liverpool).
- South West (Exeter/Plymouth).
- Southampton.
- St Mark’s Hospital, London.

Each of these Centres has demonstrated compliance with commitment 24 of the UK Strategy for Rare Diseases (https://assets.publishing.service.gov.uk/government/uploads/system/uploads/attachment_data/file/867940/dhsc-2020-update-to-the-rare-diseases-implementation-plan-for-england.pdf):

- Has a sufficient caseload to build recognised expertise.
- Service does not rely on a single clinician.
- Co-ordinates care.
- Arranges for co-ordinated transition from children’s to adult services.
- Involves people with the rare disease, and their families and carers.
- Supports research activity.

## Principles of clinical care

Patients and families with an hereditary gastrointestinal polyposis syndrome need lifelong care and support, with personalised key clinical decisions including around:

- Type and timing of colorectal surgery to prevent bowel cancer.
- Intestinal endoscopic surveillance programmes according to disease severity and underlying genetic cause of disease.
- Requirement for extra-intestinal surveillance. Patients with these conditions require lifelong care.
- Cascade genetic testing and surveillance of family members.
- Family planning/pre-implantation genetic diagnosis.

## Current situation

Currently the majority of patients and families with an hereditary gastrointestinal polyposis syndrome have care locally under non-specialists, often non-compliant with current guidelines, without sufficient experience and without offering access to research. Countless examples can be provided of suboptimal care when patients are managed outside of a structured, multidisciplinary polyposis service.

This continues despite the existence of centres of excellence, national and international guidelines, and published evidence demonstrating that outcomes are significantly improved by care in a specialist centre with dedicated call-up arrangements.

## Future provision of care

We believe that the current inequality in access to care and research is best addressed by development of the RDCN consisting of RDCC’s providing high quality specialist care, with consistent adherence to national evidence-based guidelines and standards, and collaborating in research.

Eventually all patients and families with an hereditary gastrointestinal polyposis syndrome should have their care delivered by one of these centres, with local pathways being developed to facilitate referral and patient data management systems/registries being put in place to allow effective recall, standardisation of data collection and monitoring of agreed KPI’s.

Until sufficient resource has been established within the RDCN to accommodate all patients, it is acceptable that aspects of their care (eg genetic testing, risk reduction surgery, endoscopic surveillance) be provided outside of the network, provided that current guidelines are followed and these key aspects are discussed and documented at the MDT of one of RDCC’s:

- Timing and type of risk reduction surgery.
- Endoscopic management of polyps.
- Management of extra-intestinal manifestations.

